# Antibiotic-free segregational plasmid stabilization in *Escherichia coli* owing to the knockout of triosephosphate isomerase (*tpiA*)

**DOI:** 10.1186/1475-2859-13-58

**Published:** 2014-04-21

**Authors:** Ram Shankar Velur Selvamani, Maurice Telaar, Karl Friehs, Erwin Flaschel

**Affiliations:** 1 Faculty of Technology, Fermentation Engineering, Bielefeld University, D-33594 Bielefeld, Germany

**Keywords:** Plasmid, Segregational stability, Triosephosphate isomerase, Auxotrophy, Methylglyoxal, Keio collection, Knockout strain, Continuous cultivation, *tpiA*

## Abstract

**Background:**

Segregational stability of plasmids is of major concern for recombinant bacterial production strains. One of the best strategies to counteract plasmid loss is the use of auxotrophic mutants which are complemented with the lacking gene along with the product-relevant ones. However, these knockout mutants often show unwanted growth in complex standard media or no growth at all under uncomplemented conditions. This led to the choice of a gene for knockout that only connects two essential arms of an essential metabolic pathway – the glycolysis.

**Results:**

Triosephosphate isomerase was chosen because its knockout will have a tremendous effect on growth on glucose as well as on glycerol. On glycerol the effect is almost absolute whereas on glucose growth is still possible, but with considerably lower rate than usual. This feature is essential because it may render cloning easier. This enzymatic activity was successfully tested as an alternative to antibiotic-based plasmid selection. Expression of a model recombinant β-glucanase in continuous cultivation was possible with stable maintenance of the plasmid. In addition, the complementation of *tpiA* knockout strains by the corresponding plasmids and their growth characteristics were tested on a series of complex and synthetic media. The accumulation of methylglyoxal during the growth of *tpiA*-deficient strains was shown to be a possible cause for the growth disadvantage of these strains in comparison to the parent strain for the Keio Collection strain or the complemented knock-out strain.

**Conclusion:**

Through the use of this new auxotrophic complementation system, antibiotic-free cloning and selection of recombinant plasmid were possible. Continuous cultivation and recombinant protein expression with high segregational stability over an extended time period was also demonstrated.

## Background

In 2009 almost one third (45 out of 151) of commercial biopharmaceutical recombinant proteins licensed by FDA and EMEA were produced by cultivation of *Escherichia coli*[1]. This meant that nearly 30% of high-value recombinant proteins were produced by means of a plasmid-based expression system. Various other commercial recombinant proteins (e.g. enzymes) or non-protein products (e.g. amino acids) were also obtained with strains of *E. coli* harboring plasmids. Searching the Science Citation Index database for “*coli* recombinant protein” would lead to more than 26,000 results. These facts show the high importance of *E. coli* as an expression system for commercial as well as scientific use.

Expression systems based on *E. coli* depend in almost all cases on the presence of at least one plasmid, which has to be segregated into dividing cells during growth. Insufficient segregational plasmid stability would render plasmid-based expression systems useless. Therefore, a variety of methods have been developed in order to achieve plasmid stability [2]. The commonly used method consists in adding antibiotics into the cultivation medium and placing genes for antibiotic resistance on the plasmid carrying the target gene. This strategy is widely used in research, since most often only low working volumes have to be applied. In industrial biotechnology, the addition of antibiotics can be generally excluded – not only with respect to economic reasons. The elimination of antibiotics from media and waste streams maybe required during downstream processing. Therefore, other methods for plasmid stabilization are in high demand.

A simple strategy in trying to keep a high segregational plasmid stability without adding antibiotics consists in using plasmids of high copy number and expecting that the statistical distribution of plasmids during cell division may always yield cells with at least some plasmids. This works fine as long as the average plasmid copy number is homogeneous [3]. As soon as cells with very low plasmid copy numbers show up preferentially during high growth rates, cells might have not enough time to synthesize plasmids in high copy numbers. Caused by the additional metabolic burden, plasmids may be lost completely, since plasmid-free cells would gain a growth advantage in comparison to plasmid-bearing cells. This would have a drastic influence especially on fed-batch- and continuous cultivation processes.

High metabolic burden, basal transcription levels and possible toxic effects of recombinant proteins ask for other methods for stabilizing plasmids. In some cases, changes in the cultivation strategies, mainly by decreasing the specific growth rate, may lead to sufficient plasmid stability. This can be achieved by reducing the cultivation temperature or changing the carbon source of the medium. However, most methods are based on adding some stabilizing elements by genetic engineering.

Some lessons may be learned from nature’s strategies to ensure plasmid segregation. Thus, single copy plasmids often show sophisticated stabilizing mechanisms like that based on the *par*-system which leads to a controlled distribution of plasmids, similar to the highly organized and controlled chromosome distribution in higher organisms. The *par*-system consists of at least two protein-coding genes and one special site on the plasmid for controlled distribution in the dividing cell [4].

Other systems lead to the post-segregational killing of plasmid-free cells and need the genetic information of a toxin and its corresponding antidote, with the antidote on the target plasmid [5]. Such a combination was recently applied for the development of a *Streptomyces* based protein expression system [6].

In all these cases, the plasmids may become quite large, leading to a higher metabolic burden. A larger size may also lead to another problem - decreased structural plasmid stability due to stochastic mutations, which would lead to a reduction in productivity, as well. In this regard, the *ccdB*/*ccdA* poison-antidote system, modified as a separate-component-stabilization system provides an alternative using very small genetic constructs to provide efficient antibiotic-free maintenance of plasmid in *E. coli*[7].

Many studies on DNA vaccine production have particularly been interested in alternative plasmid selection mechanisms, due to the need to avoid all kinds of resistance genes or proteins in therapeutic DNA according to safety standards. An RNA based method, using constitutive expression of *sacB* as a counter-selectable marker during growth on sucrose was reported to be able to bring about antibiotic-free selection and highly productive fermentation while not being restricted to ColE1 vectors [8].

Another perhaps more elegant way to stabilize the propagation of plasmids, is to destroy the function of an essential gene on the chromosome and place this gene on the plasmid carrying the target genes. This approach requires a strategy to generate competent cells of the now auxotrophic strain. One of the first methods for commercial use was the *valS*-system [9]. The wild-type gene for valyl-tRNA synthetase (*valS*) of the host carries a temperature-sensitive mutation, whereas the gene without mutation is placed on a plasmid. For transformation the host is grown at 30°C. During cultivations at 37°C the mutated synthetase on the chromosome loses its function and only cells carrying the *valS*-harboring plasmid can survive and grow. However, the *valS* gene is still around on the chromosome and the selection pressure favors a recombination of the mutated *valS* on the chromosome and the wild type *valS* on the plasmid. Such a recombination produces revertants with no selection pressure by *valS* and leads to plasmid instability. One way to reduce the probability of recombination is the complete knockout of the chromosomal gene. In the case of *valS* this would not allow to obtain viable cells for transformation and, therefore, would not be practicable.

The Operator-Repressor-Titration (ORT) strategy is based on negative regulation of an essential chromosomal gene by an operator sequence allowing the binding of a constitutively expressed repressor protein. In order to allow expression of the essential gene and survive, the cell has to titrate the repressor molecules against a similar operator sequence that may be present in multiple copies on the target plasmid to be maintained [10]. The essential function complementation strategy could also be effective at the RNA level. Recently, an amber nonsense mutation introduced into the essential *thyA* gene in the chromosome causing thymidine auxotrophy, was overcome by recombinant plasmids carrying a suppressor tRNA, which allowed antibiotic-free plasmid selection and also recombinant luciferase reporter expression in eukaryotic tissues and in tumour cells [11]. The fabI - triclosan system, which is an essential gene- gene product inhibitor combination, was a completely different alternative plasmid selection concept. However, the risks associated with the biocide triclosan, the requirement of the selection agent due to an addictive effect, the need to induce and over-express the essential gene marker and plasmid instability in absence of selection were also noted [12]. Another example of antibiotic-free plasmid selection is the strategy involving the essential *infA* gene coding for translation initiation factor [13].

If an auxotrophy can be overcome by supplements in the medium, competent cells can be prepared and transformed using such a supplemented medium. This may be achieved by knockout of an essential gene for e.g. the synthesis of an amino acid like glycine [14]. In this case, the *glyA* gene is knocked out in *E. coli* M15 leading to a auxotrophic strain which can be cultivated on glycine containing media. The *glyA* gene is cloned on an expression vector under the control of a constitutive weak promoter. This system has one disadvantage in that glycine-containing media may lead to plasmid-free cells, and many complex industrial media contain glycine.

One solution of this problem would consist in the construction of a knockout strain, the chromosomal knockout of which would still allow at least some growth on complex media. The specific growth rate for a strain harbouring the plasmid containing the knockout gene could generate a selection advantage high enough to keep the plasmid in the cell population.

During the search for a gene which would be appropriate for the application of the auxotrophy-based strategy for plasmid stabilization we have focused on *tpiA*, the gene for triosephosphate isomerase, a central enzyme in the glycolysis pathway. One of recent great scientific achievements in *E. coli* research was the establishment of the so-called Keio collection of knockout strains [15]. Using the Keio *tpiA* knockout strain we present the construction of *tpiA*-harbouring plasmids leading to *E. coli* strains bearing plasmids of high segregational stability.

## Results

### Screening for an appropriate knockout gene

As a consequence of biodiesel business, glycerol has evolved as an interesting carbon source for fermentation processes. A closer look on the glycerol metabolism of microorganisms, therefore, is of particular importance. *Escherichia coli* can utilize glycerol as the sole carbon and energy source. After having been imported through the cytoplasmic membrane by a facilitator protein (GlpF), glycerol can be metabolised on two alternative pathways depending on the growth conditions. One consists of a phosphorylation step by a glycerol kinase (GlpK) to yield L-glycerol-3-phosphate followed by an oxidation step due to the appropriate dehydrogenase (GlpD under aerobic and GlpABC under anoxic conditions) leading to dihydroxyacetone phosphate (DHAP). The alternative pathway consists of an oxidation step by glycerol dehydrogenase (GldA) to yield dihydroxyacetone (DHA) followed by phosphorylation by DHA kinase (DhaK) to give DHAP as well. The kinase-dehydrogenase (GlpK-GlpD/GlpABC) route is the preferred one in *E. coli*. Accordingly, different genes and their products come into play as there are: *glpF glpK glpD glpABC gldA and dhaK*. In addition, the resulting intermediate metabolite DHAP must be fed into the general glycolytic pathway through isomerisation by triosephosphate isomerase (TpiA) as glyceraldehyde-3-phosphate (GA3P). In the absence of this enzyme, DHAP is converted to the toxic compound methylglyoxal [16].

**Figure 1:**
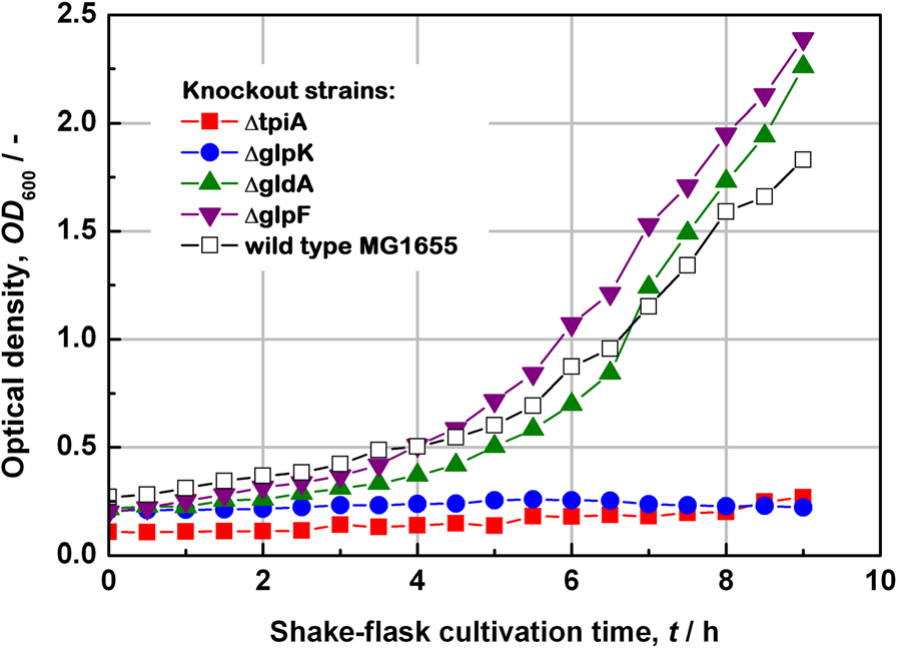
Growth testing of the wild type MG1655 and Keio deletion mutant strains in shake-flasks on a glycerol-based LB medium.

To look for an appropriate gene, four strains with a knockout of each *tpiA, glpK, gldA* or *glpF* were grown on LB-medium in the presence of glycerol as shown in Figure 1.

The knockout of *glpF*, the gene for the glycerol uptake facilitator protein, has no significant effect on growth. The normal permeability of both plasma and outer membrane may be sufficient for glycerol uptake [17]. When *gldA*, the glycerol dehydrogenase, is knocked out growth on LB medium is not disturbed, either. Compared with the growth characteristics of the *E. coli* MG1655 wild type strain, these knockouts seem to be even slightly beneficial. Keio strain Δ*glpK*, in which the glycerol kinase is knocked out, does not grow at all, although its effect might be partly circumvented [18]. The second strain showing useful characteristics is that in which the triosephosphate isomerase (*tpiA*) has been knocked out. In consequence, this gene is considered even more appropriate than *glpK* for developing a strategy for plasmid stabilization based on the auxotrophy principle since it represents an enzymatic activity essential also for the glycolysis pathway and not only for glycerol metabolism.

### Cloning of *tpiA*

For the first construct of the *tpiA* gene for complementation of *tpiA* knockout, the wild type promoter- and terminator sequences were chosen. Thus, chromosomal DNA of *E. coli* MG1655 was isolated and the *tpiA* region was amplified by PCR using 5′ · taagctggcgctatctgaatcg · 3′ and 5′ · gatggtacggcagagtgataac · 3′ as forward and reverse primer, respectively. The amplified *tpiA* fragment started 150 bp upstream of the ATG start codon and ended 172 bp downstream of the TAA stop codon of the *tpiA* structural gene. The upstream region started in the *yiiQ* gene, coding for an unknown conserved protein, including the predicted *tpiA* promoter [19]. The downstream region reached into the *cdh* gene including the predicted Rho-independent *tpiA* terminator [20]. The resulting PCR fragment of 1090 bp length was cloned into plasmid pJET (Fermentas) and subsequently verified by sequencing. This construct called pJET-tpiA was transformed into the auxotrophic host strain *E. coli* JW3890-2, CGSC#: 10805, Keio Collection (Δ*tpiA*).

All Keio knockout strains show a special structure in the genome. The knockout locations are found directly behind the ATG start codon followed by the gene for the kanamycin resistance flanked by FRT-sites. The knockouts stop 18 bases in front of the stop codon of the knocked out gene.

In case of the cloned fragment of the *tpiA* region, there was still an overlap of 153 bp upstream and 195 bp downstream of the structural target gene. This arrangement still involved the risk that a recombination between the knockout location on the chromosome and the *tpiA* region on a plasmid could occur.

### Application of the *tpiA* knockout in a recombinant production system

Previous work described the extracellular production of a hybrid bacterial β-glucanase using plasmid p582 having a size of 6 kb and a pUC19 origin of replication [21]. A strong constitutive synthetic promoter (CP7) controlled the expression of the β-glucanase gene [22]. It was followed by a *Bacillus*-derived signal sequence for periplasmic targeting. At the carboxy terminus a hexahistidine tag was placed for facilitating protein capture and detection. The other important gene on the plasmid p582 was *kil* coding for the bacteriocin release protein of ColE1 the expression of which would initiate the release of periplasmic proteins into the extracellular space. This gene was under the control of the weak stationary phase promoter of the gene *fic*. The plasmid contained antibiotic resistance genes against both ampicillin and kanamycin.

**Figure 2:**
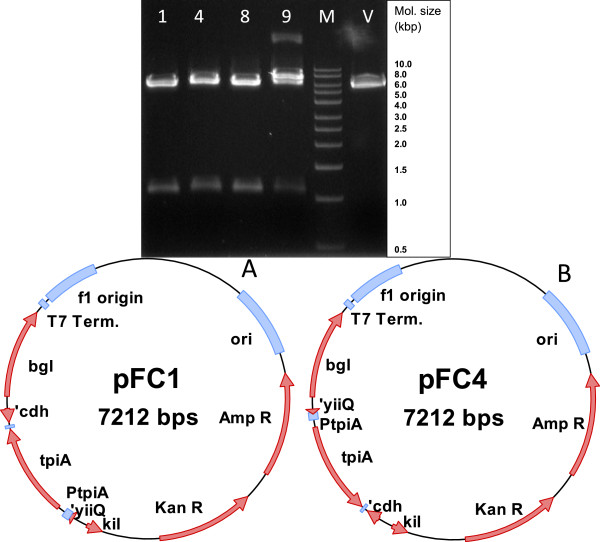
**Cloning of complementation cassette into a recombinant protein expression plasmid.** Above: EcoRI restriction and screening for positive recombinant clones. Numbers 1, 4, 8, and 9 refer to clones called pFC1, pFC4, pFC8 and pFC9. V refers to the restricted vector p582 as control. M refers to 1 kbp molecular size marker from Plasmid Factory GmbH. Below: Structure of plasmid constructs pFC1 **(A)** and pFC4 **(B)**. The constructs are each 7 kb in size and contain the recombinant β-glucanase expression gene (*bgl*) and the gene for the bacteriocin release protein (*kil*). The origin of replication (ori) is based on pUC19. The plasmids carry the beta-lactamase gene for resistance against ampicillin and neomycin phosphotransferase gene for resistance against kanamycin (shown as Amp R and Kan R respectively). The auxotrophy-complementing *tpiA* gene along with its natural promoter (PtpiA) and terminator sequences has been cloned in the forward orientation in pFC1 and in reverse orientation in pFC4.

Using the plasmid pJET-tpiA as template, the *tpiA* gene region was amplified with flanking EcoRI ends using the primers 5′ · GGAATTCtaagctggcgctatctg · 3′ and 5′ · GGAATTCgatggtacggcagagtg · 3′. This fragment was then restricted with EcoRI and cloned into the vector p582 at its unique EcoRI site. The result of the cloning procedure is shown in Figure 2. The clones 1 and 4 were subsequently verified with respect to their sequence. Partial sequencing of pFC1 and pFC4 revealed that the regions of the cloned *tpiA* gene were oriented in opposite directions.

**Figure 3:**
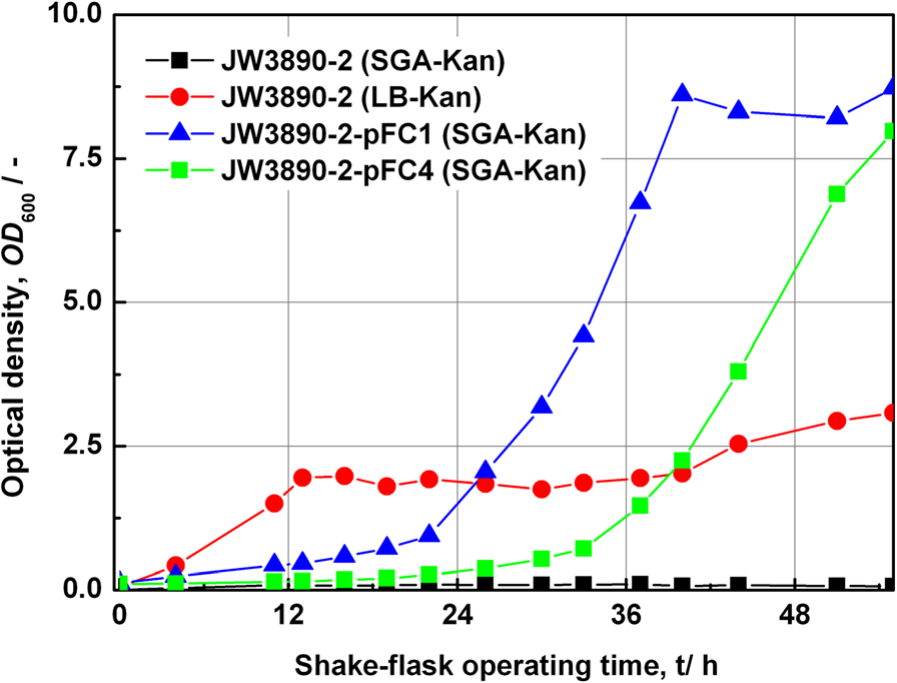
**Auxotrophy complementation of *tpiA *knockout strains.** Growth of *tpiA* knockout strain (JW3890-2) and complemented knockout strains (JW3890-2-pFC1 and JW3890-2-pFC4) were tested in different media based on glycerol only (SGA) or complex carbon sources (LB) in the presence of kanamycin in shake flasks.

The clones number 1 and 4, referred to hereafter as pFC1 and pFC4 respectively, were chosen for further studies and checked for their efficiency to complement the TpiA auxotrophy by transforming competent cells of the strain *E. coli* JW3890-2. The constructs were found to be functional and the results from growth studies in shake flasks with these plasmid constructs are shown in Figure 3.

As expected, the original Keio *tpiA* knockout strain (JW3890-2) did not grow in the presence of glycerol as the only carbon source (SGA-Kan). If a complex carbon source was present (LB-Kan), however, some growth was observed. Only the strains complemented with plasmids pFC1 (JW3890-2-pFC1) and pFC4 (JW3890-2-pFC4) showed normal growth behaviour in the presence of glycerol as the only carbon source (SGA-Kan). The strain JW3890-2-pFC1 showed a shorter lag phase while the strain with construct pFC4 had a relatively longer lag phase. In fact, this phenomenon could be reproduced over multiple trials. Both strains reached equal maximum optical densities (600 nm) of about 8.0. The media had been supplemented with kanamycin in all three cases. Although, both clones were able to complement the auxotrophy of the *tpiA* Keio knockout strain, their product expression capabilities differed considerably. Both constructs were studied under batch cultivation conditions in appropriate bioreactors.

### Batch fermentation with strain JW3890-2-pFC1

**Figure 4:**
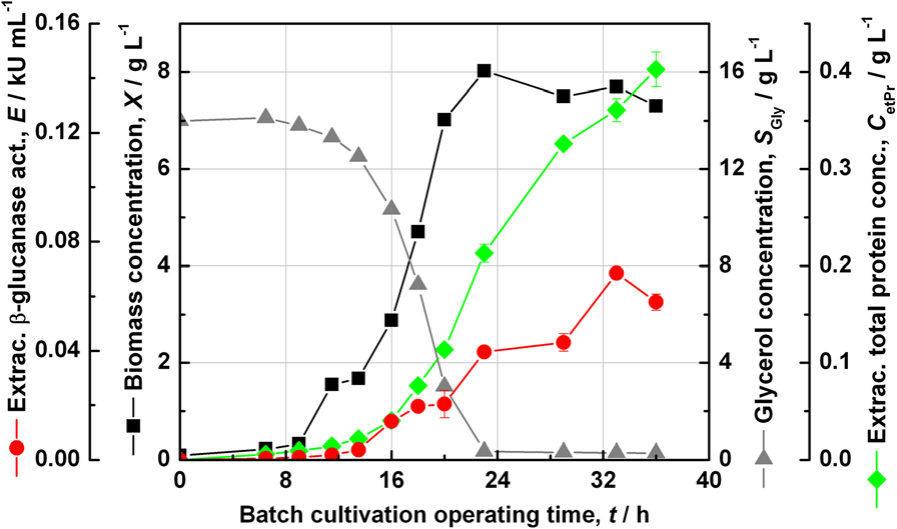
**Batch cultivation of *E. coli *strain JW3890-2-pFC1.** The Keio *ΔtpiA* knockout strain JW3890-2 complemented with the construct pFC1 was grown in SGA medium in a 2 L in-house fermenter with 1 L working volume at 30°C without the use of antibiotics.

Batch fermentation using the strain JW3890-2-pFC1 showed that the plasmid pFC1 could complement the gene knockout and enable the strain to grow with glycerol as the sole carbon source. The main results are shown in Figure 4. A biomass concentration of about 8 g L^−1^ was reached after an operating time of 24 h. Extracellular proteins appeared delayed accumulating to 0.4 g L^−1^. The extracellular concentration of β-glucanase accumulated in parallel to the profile of total extracellular protein, but reached a maximum volumetric activity of just 68 U mL^−1^. To note again, this is the construct in which the gene *tpiA* has been cloned in the forward orientation with respect to the *kil* and *bgl* gene on the plasmid p582.

### Batch fermentation with strain JW3890-2-pFC4

**Figure 5:**
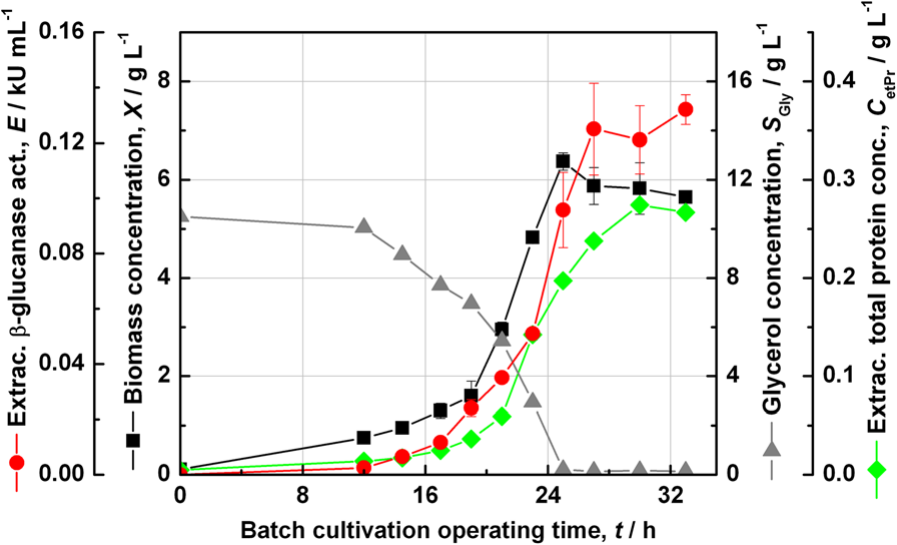
**Batch cultivation of *E. coli *strain JW3890-2-pFC4.** The Keio *ΔtpiA* knockout strain JW3890-2 complemented with the construct pFC4 was grown in SGA medium in a 2 L in-house fermenter with 1 L working volume at 30°C without the use of antibiotics.

A similar experiment was carried out with the strain JW3890-2-pFC4. The main results have been gathered in Figure 5. From these it was immediately apparent that this construct was able to achieve higher extracellular recombinant enzyme activities as compared to the other clone JW3890-2-pFC1. In fact, the maximum extracellular β-glucanase activity reached 132 U mL^−1^ at a somehow lower extracellular total protein concentration of 0.28 g L^−1^. Otherwise the time profiles were similar to those shown in Figure 4.

Since the plasmid pFC4 showed higher extracellular enzyme activity compared to pFC1, it was retained for further studies.

### Test of plasmid stabilization with *tpiA* in continuous culture

Before starting the chemostat study, the plasmid stability was tested in shake flasks by repeated subculture for over 70 generations and was found to be adequate (data not shown). As a decisive test, a chemostat was set up consisting of a small reactor of 2 L total volume in which a working volume of 1 L was maintained. The SGA minimal medium based on glycerol as the only C-source was used. The feed solution contained 20 g L^−1^ glycerol. No antibiotics were added to the media.

**Figure 6:**
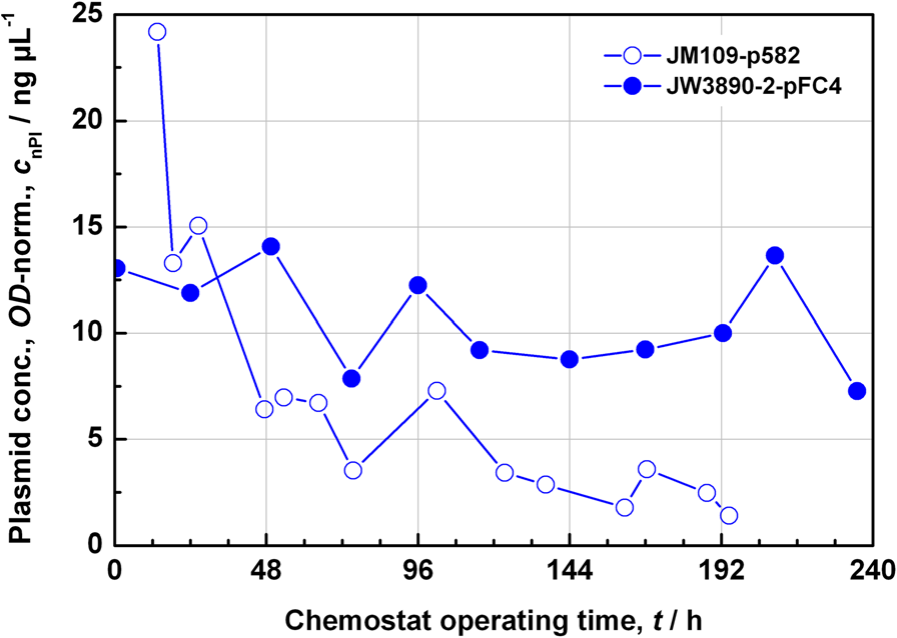
**Long-term operation of a chemostat for analysing plasmid maintenance.** Plasmid concentrations from the cells were compared for a chemostat with the control culture *E. coli* JM109-p582 and the auxotrophic system *E. coli* JW3890-2-pFC4 under antibiotic-free conditions.

At first, the chemostat was operated for an extended period of time for testing the plasmid stability. This test was not only undertaken for the complemented knockout strain JW3890-2-pFC4, but also with the control strain JM109-p582. In the latter case a constant space velocity of 0.1 h^−1^ was maintained whereas in the former case the space velocity was varied with a profile of 0.2 h^−1^ for 75 h, 0.35 h^−1^ for 21 h, 0.3 h^−1^ for 19 h, 0.1 h^−1^ for 77 h, 0.25 h^−1^ for 17 h, and 0.15 h^−1^ for an additional period of 26 h. This time profile was chosen in order to test the stability under stronger conditions than before. The results for both experiments are gathered in Figure 6. In the absence of antibiotics, the strain JM109-p582 lost its plasmid continually, whereas the plasmid concentration for the strain JW3890-2-pFC4 stabilized at about 10 ng μL^-1^. Thus, the strategy of complementing the auxotrophy based on the triosephosphate isomerase works even in the case of continuous operation. As interesting as that, the expression of extracellular proteins with *E. coli* seemed to be feasible in continuous operation as well.

**Figure 7:**
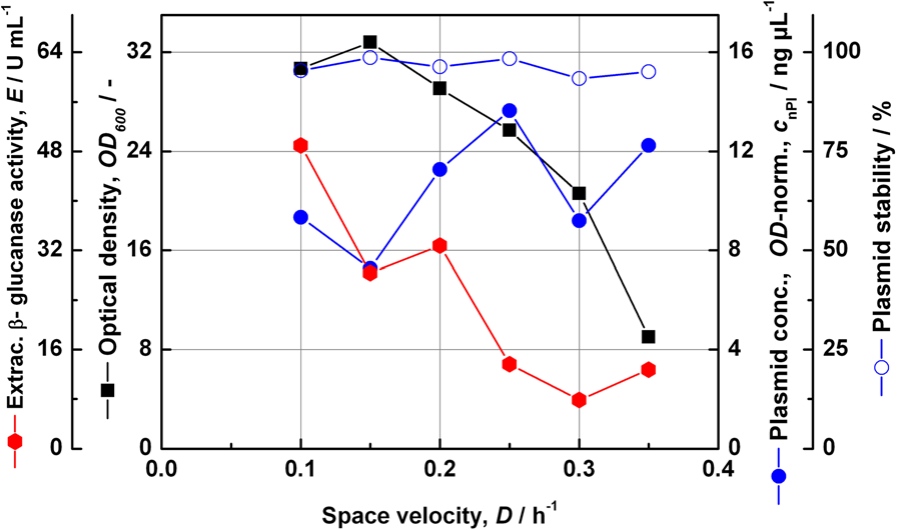
**Kinetic behaviour of the expression system JW3890-2-pFC4.** The auxotrophic complementation system was analysed by means of steady-state chemostat cultivation at different space velocities.

The chemostat experiments carried out at different space velocities offer some information about kinetic relationships. The resulting changes in cell density, extracellular β-glucanase activity, plasmid concentration and the segregational plasmid stability were analysed and the results obtained are shown in Figure 7. The decline of the cell density with higher space velocities revealed a pattern of kinetics far from exponential growth. A similar unfavourable characteristic was found for the extracellular enzyme activity the decline of which was rather linear as a function of space velocity. The maximum volumetric enzyme productivity was fairly low at about 6.5 U mL^−1^ h^−1^. The plasmid stability by replica plating was almost 100% through the whole process.

### Construction of a *tpiA* vector with altered downstream overlap

In order to decrease the possibility of recombination by the overlaps of the chromosome base sequence and the *tpiA* region on the plasmid, the original *tpiA* terminator of pJET-tpiA was replaced by an artificial bidirectional terminator leading to the plasmid pJET-tpiA-Tart. This terminator had the sequence AAAAAAAAAAAGGGGCGAAGCCGCCCCTTTTTTTTT and was cloned downstream of the *tpiA* gene on pJET-tpiA using the primers shown in Table 1. The portions forming the terminator have been shown in upper case letters.

**Table 1:** Primers for cloning an artificial terminator behind the tpiA gene on pJET-tpiA

**Primer name**	**Sequence**
*tpiA* Term. lacUV	5′-CGCCCCTTTTTTTTTagcctggggtgcctaatgag-3′
*tpiA* Term. direct	5′-GCTTCGCCCCTTTTTTTTTTTaagcctgtttagccgcttctgc-3′

### Growth of the *tpiA* knockout strain on different media in the absence or presence of the complementing plasmid pJET-tpiA-Tart

**Figure 8:**
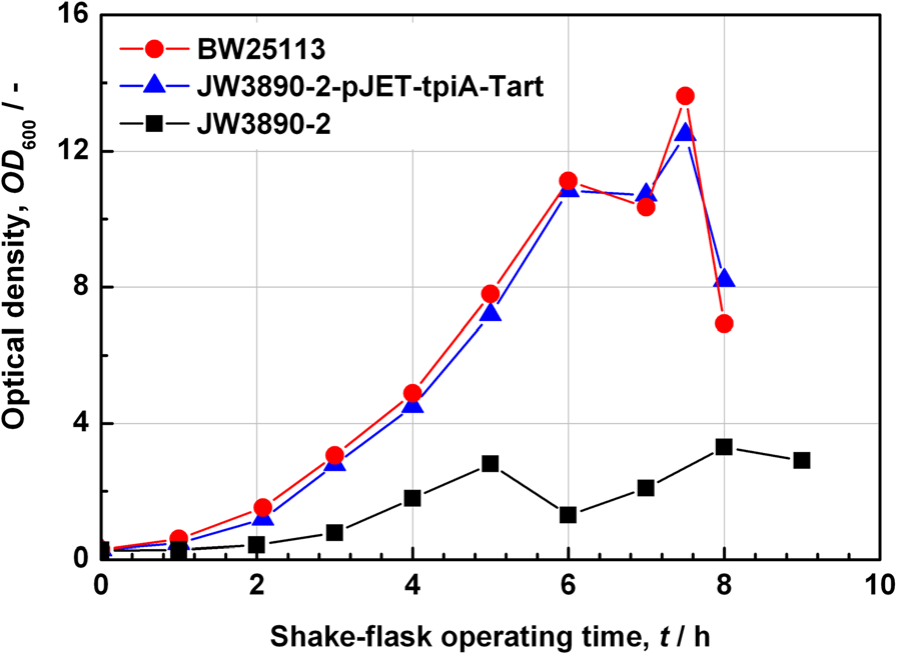
**Growth of the parent strain for the Keio Collection BW25113 and JW3890-2 deletion mutant strains.** The mutant strains were grown in the absence or presence of the complementing plasmid pJET-tpiA-Tart. The cultivations proceeded in shake flasks on the glucose-based HSG medium.

The first step in growth analysis of the *tpiA* systems was to determine the growth rates of the parent strain for the Keio Collection *E. coli* BW25113, the Keio deletion mutant JW3890-2, and the plasmid-supplemented mutant strain. In all these experiments the *tpiA* deficient strain showed lower maximal optical densities and lower growth rates than the parent strain for the Keio Collection and the plasmid supplemented *tpiA* deficient strain JW3890-2. As an example the growth on the glucose-based HSG medium is shown in Figure 8.

**Figure 9:**
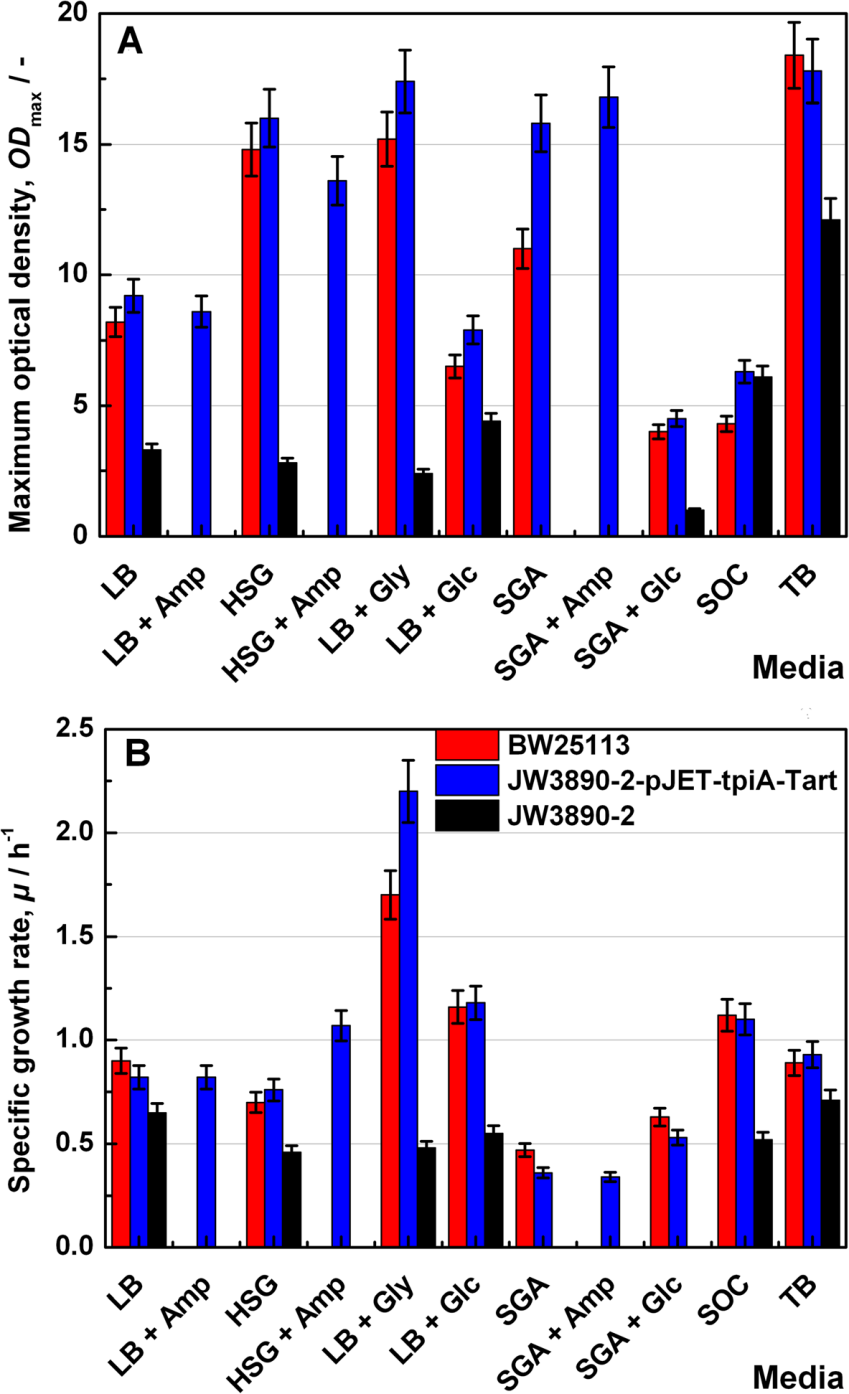
**Influence of medium composition on the growth kinetics.** Maximum biomass concentration (*OD*_600_) **(A)** and the specific growth rate (μ) **(B)** of *E. coli* Keio strains: BW25113, knockout mutant in the presence (JW3890-2-pJET-tpiA-Tart), and in the absence (JW3890-2) of the complementing plasmid. The legend applies to both Figure parts (A and B).

The Keio *tpiA* knock out strain JW3890-2 reached the stationary phase in HSG medium early. Other media tested were LB, LB + ampicillin, HSG + ampicillin, LB + glycerol, LB + glucose, SGA, SGA + ampicillin, SGA + glucose, SOC and TB, the terrific broth. The composition of the media applied is to be found under Methods. As shown in Figure 9, the *tpiA* knock out strain JW3890-2 showed no growth in synthetic media. In all other media the strain showed lower growth rates than the parent strain for the Keio Collection and the plasmid bearing strain.

The growth rates of the parent strain for the Keio Collection and the plasmid carrying strain were quite similar. The plasmid supplemented strain was able to grow in synthetic media, whereas the plasmid-free *tpiA* deletion strain JW3890-2 was not able to grow on synthetic media.

### Analysis of plasmid stability

Because of the fact that the plasmid free *tpiA* deletion strain could not grow on synthetic media, the experiments for antibiotic free selection were performed in SGA medium. For this purpose shake-flask cultivations were carried out in a repetitive fashion by reintroducing 100 μL of a final cultivation broth into 50 mL of fresh medium in shake flasks of 500 mL for a cultivation period of 1 day. The same procedure was applied to the parent strain for the Keio Collection as well as to the mutant Δ*tpiA* Keio strain in the presence of either pJET-tpiA or pJET-tpiA-Tart as complementing plasmid. For each series 13 repeated cultures were followed over a total period of 17 d.

For a cultivation time of 1 d, the parent strain for the Keio Collection strain grew to an optical density of 8.1 ± 0.25 whereas the pJET-tpiA complemented mutant strain reached 12.1 ± 0.53, and that complemented with pJET-tpiA-Tart grew to an OD_600_ of 11.3 ± 0.57. The data are given with their standard deviations collected over 10 samples. The segregational plasmid stability was tested at the same time and was found to be stable at 98 to 100%.

In addition, the plasmid stability was tested for cultivations in the presence of different media in the absence of ampicillin. Thus, the plasmid maintenance was found to be absolute for the growth of the Keio mutant strain complemented with pJET-tpiA-Tart in the case of the media HSG, LB, LB supplemented with glucose, LB supplemented with glycerol, SGA, SGA supplemented with glucose, SOC as well as TB.

Because of these data it can be expected, that the TpiA deficiency can be used as a selection marker for plasmid stabilization.

### Accumulation of methylglyoxal may lead to a toxic effect

The reason why the TpiA deficient strain stopped growing at rather low biomass concentrations compared with the wild type or the plasmid-supplemented strain during cultivation on different media as shown in Figure 9 could be a consequence of DHAP accumulating in the cells. This could in turn lead to methylglyoxal by enzymatic conversion of DHAP by MgsA (methylglyoxal synthase), which should be toxic for the cells. Some experiments were performed in order to test this assumption.

**Figure 10:**
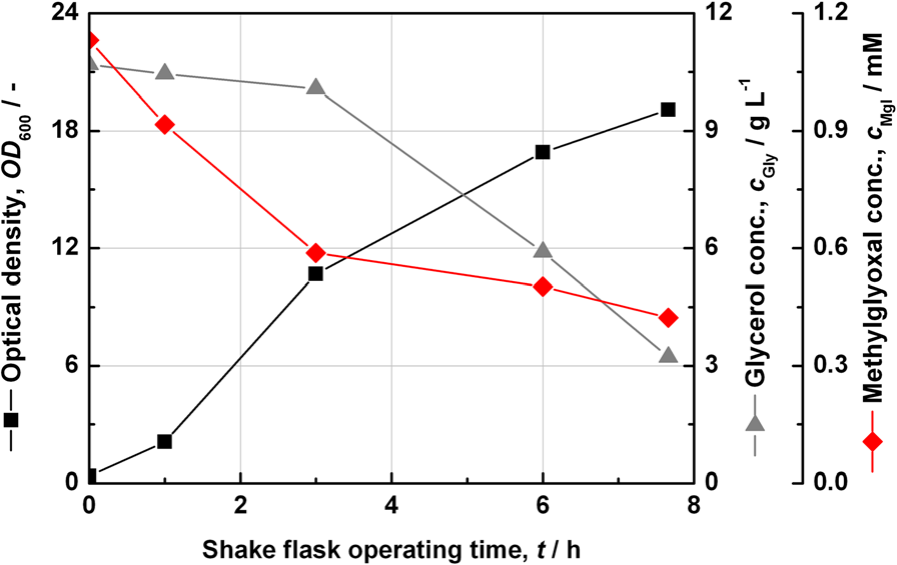
**Determination of the toxic effect of methylglyoxal.** Shake-flask cultivation of *E. coli* JW3890-2-pJet-tpiA-Tart on spent HSG medium obtained by prior growth of JW3890-2 on HSG medium.

Thus, the *tpiA* deficient strain JW3890-2 was cultivated in HSG medium overnight. An optical density (OD_600_) of 4.3 was obtained. The culture was centrifuged and the supernatant was sterile filtered. The medium still contained 10 g L^−1^ glycerol (initial concentration was 14.9 g L^−1^). In parallel, a culture of JW3890-2-pJet-tpiA-Tart had been cultivated in HSG medium overnight. The sterile filtered medium was inoculated with JW3890-2-pJet-tpiA-Tart at an initial optical density (OD_600_) of 0.35. The concentration of glycerol and methylglyoxal were analysed in addition to the optical density along the cultivation process. Figure 10 shows the results. The plasmid supplemented strain grew to an optical density (OD_600_) of 20 within 8 hours by consuming 7 g L^−1^ glycerol.

The data show that methylglyoxal accumulated during the cultivation of the *tpiA* deficient strain and that it was consumed during the second cultivation on the spent medium, and fell from 1.1 mM to 0.4 mM. A methylglyoxal concentration of 0.6 mM is assumed to be toxic for *E. coli*[16]. Thus, the toxicity of methylglyoxal generated from DHAP may force an auxotrophic effect even in the presence of glucose as the main carbon source.

### Antibiotic free cloning

To test the possibility of antibiotic free cloning with *tpiA* deficient strains, SGA agar plates were created by adding 15 g L^−1^ agar agar into SGA medium and pouring into petri dishes. The *tpiA* deficient strain was exposed on these plates and showed no growth. After transforming the plasmid pJET-tpiA-Tart in a chemical competent JW3890-2 strain, colonies appeared in the antibiotic free SGA plates containing the correct plasmid.

## Discussion

The constructs pFC1 and pFC4 contain the auxotrophy-complementing *tpiA* gene in opposite orientations and some consequences of this difference could be observed from Figures 4 and 5. With respect to growth, it was seen over multiple trials that the strain JW3890-2-pFC1 grew with a slightly shorter lag phase and achieved higher maximum biomass concentrations than JW3890-2-pFC4. Whereas, with respect to the extracellular recombinant β-glucanase activity, the construct pFC4 clearly returned higher volumetric activity than pFC1 and this difference could also be reproduced over multiple trials. It is common for expression levels to vary between different clones and in this case one possible scenario is that in the case of pFC1, the *tpiA* gene could be read into the downstream product gene *bgl*, which has the same orientation.

Other experiments conducted to check the secretion achieved with these constructs showed that both the clones pFC1 and pFC4 were poor in secretion into extracellular space. The analysis of samples from the stationary phase showed that, the activities in the periplasmic fraction were higher than those in the extracellular fraction. This was in clear contrast to the control strain *E. coli* JM109-p582, where the reverse was to be seen and the equilibrium was clearly in favour of the extracellular fraction (data not shown). In general, the control strain showed up to 10 times the activity achieved with the auxotrophic strains and thus represented an ideal case. However, since the auxotrophic system offers antibiotic-free cultivation, it deserves further development. The question of what could be causing the suboptimal activity of the BRP in the Keio strains remains open at this moment. The general low efficiency of these auxotrophic hosts could be one possible explanation. In this regard, *tpiA* gene knockouts from the control strain are being developed which should then be able to serve as efficient hosts for the expression and secretion of the recombinant product.

As mentioned under the section Results, the target gene deletion in the Keio strains is restricted to the region between the start codon and the last six codons. The gene deletion in the control strain would be wider and more extensive than in the Keio strain and is thus designed to overcome the risk of recombination of the plasmid-borne *tpiA* copy with the host genome and loss of auxotrophy. Another potential route to better productivity from the clones would be the expression of *tpiA* at the minimum required level. This would avoid unnecessarily high rates of transcription and stress in the cells. Therefore, the native P1/P2 promoters for *tpiA* are being replaced with artificial weak promoters from a synthetic promoter library [23].

Using the Keio knockout strains, a flux from DHAP into the xylulose pathway is not possible due to the deletion of rhamnose metabolism genes [15]. A bypass of DHAP to lactate and pyruvate through methylglyoxal is possible through the glyoxalase I-II pathway [24]. This should also explain the limited growth of cells without the complementation plasmid in complex medium. However, when glycerol is the sole carbon and energy source, the toxicity of methylglyoxal should render this route unfavourable. Moreover, this would only be a partial metabolism and not an energy-efficient pathway to give a growth rate advantage.

## Conclusions

Antibiotic-free recombinant plasmid selection and stabilization in *E. coli* based on the auxotrophy complementation of the activity of TpiA has been demonstrated. From our observations, we have been able to achieve antibiotic-free cloning, selection, expression of a model recombinant product and long-term stability of the plasmid in continuous culture. The growth advantage shown by the plasmid-complemented strain even under non-selective conditions makes this system particularly attractive for large-scale industrial processes.

## Methods

The list of strains of *E. coli* used in this study is shown in Table 2.

**Table 2:** **List of *Escherichia coli *** **strains used**

***E. coli *** **strain**	**Genotype**	**Source**
BW25113 (parent strain for the Keio Collection)	Δ*(araD-araB)567*, Δ*lacZ4787*(::rrnB-3), lambda^−^, *rph-1*, Δ*(rhaD-rhaB)568*, *hsdR514*	CGSC, Yale University
JW3890-2	Δ*(araD-araB)567*, Δ*lacZ4787*(::rrnB-3), lambda^−^, *rph-1*, Δ*(rhaD-rhaB)568*, *hsdR514 ΔtpiA778::kan*	CGSC, Yale University
MG 1655	F^−^, lambda^−^, rph-1	DSMZ
JM109	*endA1*, *recA1*, *gyrA96*, *thi*, *hsdR17* (rk–, mk+), *relA1*, *supE44*, Δ(*lac-proAB*), [F’ *traD36*, *proAB*, laqI^q^ZΔM15]	Promega

All the modifications in the knockout strain other than the *tpiA* deletion are already present on the parent strain *E. coli* BW25113. The modifications in the arabinose, lactose or rhamnose metabolic genes do not have any consequences for the current experiments reported with this strain.

The plasmids applied for the experiments described are gathered in Table 3.

**Table 3:** List of plasmids used

**Plasmid**	**Features/Genes**	**Source**
p582	rep (pUC19), *bla* (Amp^R^), *npt* (Kan^R^), P_CP7_-*bgl*, P_fic_-*kil*, T7 terminator, multiple cloning site	[21]
pJET 1.2	rep (pMB1), *bla* (Amp^R^), *eco47IR*, P_lacUV5_, T7 promoter, multiple cloning site, insertion site	Fermentas
pJET-tpiA	rep (pMB1), *bla* (Amp^R^), *eco47IR*, P_lacUV5_, T7 promoter, residual multiple cloning site, *tpiA* expression cassette	This work
pFC1	rep (pUC19), *bla* (Amp^R^), *npt* (Kan^R^), P_CP7_-*bgl*, P_fic_-*kil*, T7 terminator, *tpiA* expression cassette in forward orientation	This work
pFC4	rep (pUC19), *bla* (Amp^R^), *npt* (Kan^R^), P_CP7_-*bgl*, P_fic_-*kil*, T7 terminator, *tpiA* expression cassette in reverse orientation	This work
pJET-tpiA-Tart	rep (pMB1), *bla* (Amp^R^), *eco47IR*, T7 promoter, residual multiple cloning site, *tpiA* expression cassette, artificial terminator	This work

The media applied for growth experiments with *E. coli* strains are shortly explained prior to presenting their exact composition in Tables 4 and 5 for convenience.

**Table 4:** Composition of the SGA medium

**Solution name**	**Volume fraction/%**	**Component**	**Solution concentration/g L**^**−1**^	**Medium concentration/g L**^**−1**^
Micronutrient solution 500×	0.2	FeCl_3_ **·** 6H_2_0	5.4	0.0108
		ZnSO_4_ **·** 7H_2_O	1.38	0.00276
		MnSO_4_ **·** H_2_0	1.85	0.0037
		CoSO_4_ **·** 7H_2_0	0.56	0.00112
		CuCl_2_	0.17	0.00034
		H_3_BO_3_	1	0.002
		Na_2_MoO_4_ **·** 2H_2_O	2.5	0.005
		Citric acid hydrate	5	0.01
Salt solution 10×	10	K_2_HPO_4_	135.3	13.53
		KH_2_PO_4_	66.2	6.62
		Citric acid hydrate	18.4	1.84
		EDTA	0.08	0.008
Glycerol solution	1	Glycerol	1,000	10
Glucose solution	10	Glucose	195.6	19.56
Ammonium sulphate solution	1.2	(NH_4_)_2_SO_4_	500	6
MgSO_4_ solution	0.24	MgSO_4_	250	0.6

**Table 5:** Composition of the HSG medium

**Medium component**	**Medium concentration/g L**^**−1**^
Soy peptone	13.5
Yeast extract	7
Glycerol	14.9
NaCl	2.5
K_2_HPO_4_	2.3
KH_2_PO_4_	1.5
MgSO_4_ **·** H_2_0	0.14

SGA media are fully synthetic ones. All solutions were autoclaved separately and combined as explained in Table 4. Depending on the preferred carbon source either glycerol or glucose was added. For batch fermentations with SGA, water and carbon source were autoclaved *in situ*, while the other components were sterilized separately and added to the reactor. When the medium was complete, the pH was found to be around 6.5. This was then corrected to a pH of 7 through addition of sterile NaOH before inoculation. For the strain JM109, sterile-filtered thiamine solution was added at a final concentration of 0.01 g L^−1^ due to its auxotrophy as given in the genotype.

The HSG medium is a buffered complex rich medium with glycerol as carbon source. The K_2_HPO_4_/KH_2_PO_4_ solution was autoclaved separately. The pH was adjusted to 7.4. Its composition is explained in Table 5.

Other media like LB (Lysogeny Broth), TB (Terrific Broth) and SOC (Super Optimal broth with Catabolite repression) were prepared according to standard laboratory protocols.

### Cultivation

#### Shake flask cultivation

All cultivations were performed in a working volume of 50 mL in shake flasks of a total volume of 500 mL equipped with baffles (Schott, Germany). The rotary shaker LS-X (Kühner, Switzerland) had a rotating frequency of 120 min^−1^ and an eccentricity (diameter) of 50 mm. The cultivation was carried out at a temperature of 30°C.

#### Bioreactor

The bioreactor cultivations were carried out in a 2 L in-house fermenter with a working volume of 1 L. The reactor had a height of 280 mm and a diameter of 94.4 mm. The impeller diameter measured 46 mm and the impeller blades measured 12 mm on each side. A total of 3 impellers with 6 blades each ensured good mixing for homogeneous conditions. Moreover, the vessel contained 4 baffles with a height of 260 mm and width of 8 mm each.

#### Batch cultivation

Water and glycerol for the SGA medium were sterilized *in situ* along with the fermenter, while the remaining components were sterilized separately and added under sterile conditions. After adjusting initial pH and saturating the medium with oxygen, the fermenter was inoculated from an overnight shake flask culture. Agitation was carried out at a stirrer speed of 800 rpm and aeration was achieved with an air space velocity of 1 vvm. The process temperature was maintained at 37°C for the control strain *E. coli* JM109-p582 and 30°C for the auxotrophic strains, since the latter showed inclusion body formation due to stress (data not shown) when grown at 37°C. The pH was automatically maintained at 7.0 using 10% orthophosphoric acid and 2 M sodium hydroxide as correction solutions. The process was monitored by a ADAM-4060 relay output module (Advantech Ltd., USA) and run by using DASYLAB 6.0 software (National Instruments Service GmbH, Germany).

#### Continuous cultivation

The chemostat process was started as a batch fermentation, and upon reaching the end of exponential phase, the feed and outlet pumps were switched on. The desired space velocity (dilution rate) was set by the flow rate of the medium. The feed medium supplied from a 40 L reservoir had the same composition as the medium for batch but with 20 g L^−1^ glycerol. The culture volume in the reactor was maintained at a constant level by operating the inlet pump at the exact flow rate required whereas the outlet pump was operated at twice the flow rate, with the suction pipe entrance positioned at a fixed depth corresponding to a liquid working volume of 1 L. Stably maintained levels of dissolved oxygen (DO) in the reactor medium, CO_2_ content in the exit air and optical density of the culture indicated a steady state. Any change in the feed flow rate was followed by allowing the system to stabilize by passing at least three to four culture volumes through the reactor before a new steady state was assumed.

### Measurement of growth

Prior to each cultivation experiment a single colony was transferred from an agar plate into the appropriate medium and cultivated overnight. The pre-culture media were exactly the same as the media used in the subsequent cultivation experiments.

For the main cultivation the initial optical density at a wavelength of 600 nm (*OD*_600_) was adjusted to 0.1. Over the cultivation process samples were taken and the optical density was measured against the cultivation medium. If required, samples were diluted with fresh cultivation medium.

### Determination of the specific growth rate

The specific growth rate (μ) was determined by means of the standard method [25].

### Plasmid stability

The plasmid stability was determined by plating out appropriately diluted samples on normal LB agar plates prepared without antibiotics and incubating at 37°C for at least 15 h. The colonies were then transferred onto an LB agar plate containing 200 mg L^−1^ ampicillin followed by incubation at 37°C for at least 15 h. The ratio of the number of colonies counted on plates prepared in the presence of antibiotics to that observed in the absence of antibiotics was interpreted as plasmid stability.

For measurements of the plasmid content in the samples, a fixed volume of culture broth (2 mL) was used for plasmid isolation by the Wizard Plus SV DNA Purification System (Promega, USA). The elution of the plasmid from the column was carried out into a constant volume (50 μL) of nuclease-free water (Promega, USA). The plasmid DNA concentration was measured by means of the Nanodrop spectrophotometer (Peqlab Biotechnologie GmbH, Germany), and the value was normalised to the optical density of the culture sample.

### Determination of methylglyoxal concentration

Methylglyoxal was quantitatively determined according to the method described by Cooper [26].

### Determination of glycerol concentration

Culture samples were centrifuged to separate the cells. Glycerol concentration in the supernatant was measured by HPLC using a Nucleogel sugar 810H cation exchange column (Macherey-Nagel GmbH, Germany).

### Assay for β-Glucanase activity

The recombinant protein expression was analysed with an activity test for endo-1,3-1,4-β-glucan-glucanohydrolases. Substrates used were either lichenan (Roth, Germany) or barley β-glucan (Megazyme, Ireland) both of which have very similar properties. The substrate contains 70% β-1,4- and 30% β-1,3-bonds.

The substrate solution in 40 mM sodium acetate buffer [27] was incubated with samples containing the enzyme to be assayed for 20 min. The reducing groups released were analysed by the method based on dinitrosalicylic acid by following the absorbance of light at a wavelength of 530 nm (*OD*_530_). This was compared to the maximum value expected for the particular substrate at complete conversion, which can be obtained practically by applying an excess enzyme activity and incubating for 2 h. The final enzyme activity was obtained as a volumetric activity taking into account the particular enzyme kinetics [27]. One unit is defined as the enzyme activity that releases an equivalent amount of 1 μmol glucose residues per min at a temperature of 50°C and a pH 5.6.

### Cloning

All cloning experiments were carried out according to standard methods [28].

### Sequencing

Potential clones were verified by sequencing the cloned region using corresponding sequencing primers. Cycle-sequencing was performed at the Centre for Biotechnology (CeBiTec) (Bielefeld University, Germany). A BigDye® terminator version 3.1 chemistry was used for the PCR in a GeneAmp® PCR System 9700 (both Applied Biosystems, Germany). The fragments of various lengths were then separated by capillary electrophoresis and the fluorescence signal of each base converted to their corresponding digital data by a 96-well 3730xl DNA Analyzer (Applied Biosystems, Germany).

## Competing interests

The authors declare that they have no competing interests.

## Authors’ contributions

RSVS and MTE designed and performed the experiments, analyzed and interpreted the data. KF and EF wrote the manuscript. KF and EF conceived the study and supervised the research. All authors read and approved the final manuscript.
